# An Image-Based Algorithm for Precise and Accurate High Throughput Assessment of Drug Activity against the Human Parasite *Trypanosoma cruzi*

**DOI:** 10.1371/journal.pone.0087188

**Published:** 2014-02-04

**Authors:** Seunghyun Moon, Jair L. Siqueira-Neto, Carolina Borsoi Moraes, Gyongseon Yang, Myungjoo Kang, Lucio H. Freitas-Junior, Michael A. E. Hansen

**Affiliations:** 1 Image Mining (IM) Group, Institut Pasteur Korea, Seongnam-si, Gyeonggi-do, South Korea; 2 Center for Neglected Diseases (CND3), Institut Pasteur Korea, Seongnam-si, Gyeonggi-do, South Korea; 3 Chemical Biology of Pathogen (CBP) Group, Institut Pasteur Korea, Seongnam-si, Gyeonggi-do, South Korea; 4 Department of Mathematics, Seoul National University (SNU), Gwanak-Gu, Seoul, South Korea; Federal University of São Paulo, Brazil

## Abstract

We present a customized high content (image-based) and high throughput screening algorithm for the quantification of *Trypanosoma cruzi* infection in host cells. Based solely on DNA staining and single-channel images, the algorithm precisely segments and identifies the nuclei and cytoplasm of mammalian host cells as well as the intracellular parasites infecting the cells. The algorithm outputs statistical parameters including the total number of cells, number of infected cells and the total number of parasites per image, the average number of parasites per infected cell, and the infection ratio (defined as the number of infected cells divided by the total number of cells). Accurate and precise estimation of these parameters allow for both quantification of compound activity against parasites, as well as the compound cytotoxicity, thus eliminating the need for an additional toxicity-assay, hereby reducing screening costs significantly. We validate the performance of the algorithm using two known drugs against *T.cruzi*: Benznidazole and Nifurtimox. Also, we have checked the performance of the cell detection with manual inspection of the images. Finally, from the titration of the two compounds, we confirm that the algorithm provides the expected half maximal effective concentration (EC50) of the anti-*T. cruzi* activity.

## Introduction

Chagas disease is a tropical neglected disease caused by the flagellate protozoan *Trypanosoma cruzi*, transmitted to humans by the Triatominae insects (kissing bug), by the ingestion of food contaminated with live forms of the parasite, or through contaminated blood transfusion and organ donation. Chagas disease is endemic in Latin America, where it is estimated to affect 10 million people [1]. The disease manifestation can range from asymptomatic to flu-like fever in the acute stage, and life-threatening heart and digestive system disorders in the chronic stage, years after the beginning of infection. Due to global trends of migration, massive numbers of infected individuals have carried and transmitted the parasite to non-endemic regions such as North America, Europe, Japan and Australia especially through blood transfusion [2]. The only two available chemotherapies are Benznidazole and Nifurtimox, showing both high toxicity with severe side effects and being sometimes ineffective [3]. Therefore, new drugs are urgently needed to treat Chagas disease.

The screening of large collections of chemical compounds is one initial step towards the discovery of a new and better treatment. In this aspect, high content screening (HCS) technologies have advanced the discovery of new chemical entities to treat neglected diseases [2]–[7]. Automated image acquisition technology and computerized image mining techniques can provide unique multi-parametric and highly accurate information of chemical compounds activity against the intracellular parasite, enabling the implementation of high-throughput experimentation [4].

We describe a fully automated image analysis algorithm for HCS in anti-trypanosomal drug discovery. The algorithm is capable of interpreting the infection and quantifying the activities of the anti-parasitic compounds by precise detection of parasite and host cell nuclei, as well as host cell cytoplasm in the images. Additionally the algorithm can estimate the compound cytotoxicity over host cells by counting the total number of cells in the acquired images, thus eliminating the need for secondary assays to assess compound cytotoxicity and determining the selectivity of a compound. We compared the algorithm by comparison with manual inspection of fluorescence images of a Draq5 stained human cell line infected with *Trypanosoma cruzi*. This comparison have shown that the difference of the segmented nuclei numbers from the algorithm and manual inspection results was consistently less than 5%. Finally, dose-response curves (DRC's) with Benznidazole and Nifurtimox demonstrated that the algorithm was capable of precisely detecting both infected host cells and their intracellular parasites.

## Materials and Methods

### Parasite Culture, Image Acquisition Process and Image Analysis Algorithm

The experimental assay was set up by the following protocol: The U2OS human cell (HTB-96, ATCC, Manassas, VA) was used for the *in vitro* assay preparation and the cells were infected with wild type *T. cruzi* and GFP-tagged *T. cruzi* Tissue Culture Trypomastigotes (TCT) [8]. Metacyclic trypomastigotes were obtained from a late stage epimastigote in order to generate this stage of parasites. The metacyclic trypomastigotes were used to infect the LLC-MK2 cell line (CCL-7, ATCC, Manassas, VA). Seven days after the infection, supernatant containing TCT parasites was collected and used to re-infect new cultures of LLC-MK2. Parasites from days 6, 7 and 8 were collected from the supernatant of the LLC-MK2 infected culture and used to infect U2OS in the assay plates. Re-infection was performed with parasites from the 7th day after the previous infection. To perform the *in vitro* infection in U2OS, cells and parasites were mixed in DMEM-Low glucose media, supplemented with 2% heat-inactivated Fetal Bovine Serum. This homogeneous mixture of U2OS cells and parasites was seeded into the 384-well plates at 50 *µ*l/well and incubated for 48 hours at 37°C, 5% CO_2_. After incubation was done, 12% paraformaldehyde (PFA) in PBS solution was added and 80 *µ*l of media was removed using EL406 BioTek automated liquid handler. The cells and parasites were stained with 5 *µ*M Draq5 (Biostatus) in 4% PFA solution. Infection with GFP-tagged *T.cruzi* was performed same way as wild type *T.cruzi*. Images are taken at 635 nm and 488 nm excitation filter in Operetta imaging system.

Following the host cell and the parasite staining as described above, four microscopic image fields (of 1360×1024 pixel^2^  = 680×512 *μ*m^2^) were acquired from each assay well using an automated Operetta 2.0 imaging system (Perkin Elmer) with conditions of 635 nm filter of 90% excitation, 20× lens magnification, 0.45 numerical aperture, 300 ms exposure time and non-confocal optic mode. The acquired images were transferred to a central image database in real-time and saved as 16-bit TIFF format (unsigned integer of range 0 to 65535). The stored images were accessed through an in-house developed image analysis software platform named Image Mining (IM). This platform serve as an interface between the central image database (from the Operetta) and the dedicated image analysis algorithm doing the image analysis, and can process large amounts of image data generated [9]. The developed algorithm was implemented as a ‘plug-in’ to the IM platform, so that is able to use the automated analysis capability of the IM platform.

Since Draq5 was used to label DNA of both host cells and *T. cruzi* parasites, host cell and parasite information were mixed into one single channel. Image properties can be summarized as:

1. The intensity range of the nuclei and parasites was 1400 to 1800, and cytoplasm was 300 to 500.
2. Host cell nuclei were typically clustered or very close together.
3. There were objects of heterogeneous high intensity such as apoptotic cells or cells under division with condensed DNA.
4. The images were often subject to an illumination bias, which occurred during the image acquisition process.

**Figure 1:**
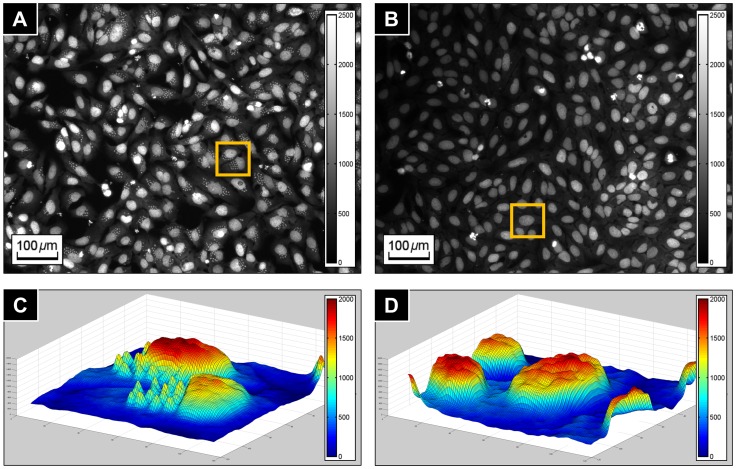
*T. cruzi* fluorescence images. (**A**) Negative control image (infected host cells). (**B**) Positive control image (uninfected host cells). (**C**) 3D surface plot of infected host cells in the yellow boxed region of (A). (**D**) 3D surface plot of uninfected host cells in the yellow boxed region of (B). Note that intensity ranges of (A) and (B) are rescaled for enhanced visibility.

Figure 1 shows a control image of infected and untreated host cells (shown in (A)), a control image of uninfected host cells (shown in (B)), a 3D surface plot of an infected host cell (shown in (C)) and uninfected host cell (shown in (D)). Note that the intensity scale of negative and positive control images in Figure 1 have been changed for illustrational purpose. The original images of Figure 1A and 1B are presented in Figure S1 and S2 respectively.

Based on the above properties, the algorithm segmented regions containing nuclei, identified clustered nuclei and split them into individual nuclei, segmented cytoplasm, and finally detected parasites. In short, this algorithm consisted of five major parts (Figure S3):

1. Image enhancement by intensity equalization and shading correction.
2. Nuclei region segmentation by a new method based on the Laplacian of Gaussian and morphological operations.
3. Individual nuclei segmentation by the gradient flow tracking segmentation.
4. Cytoplasm segmentation by the seeded cell segmentation.
5. Parasites detection by the local extreme detection method.

### Image Enhancement Process

Before entering the main part of the algorithm, raw images of *T. cruzi* go through a two-step image-enhancement process in order to obtain more reliable results.

**Figure 2:**
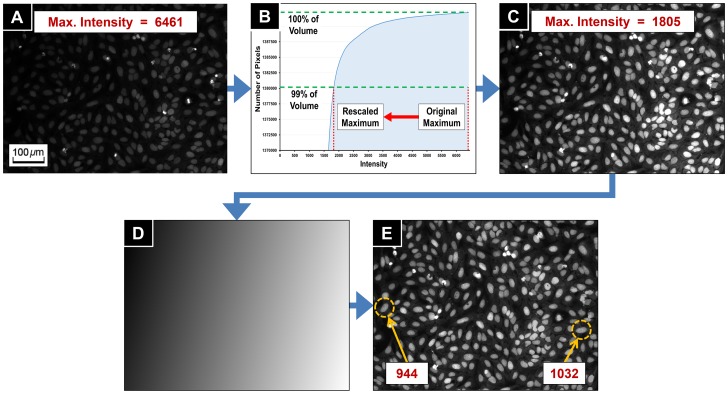
Image enhancement process. (**A**) Original image of maximum intensity 6461. (**B**) Cumulative histogram of intensity. The maximum intensity is rescaled to the intensity of higher 1% cumulative intensity level. (**C**) Intensity equalized image. The maximum intensity has decreased to 1805. (**D**) Estimated shading map of (C). (**E**) Biased illumination was corrected from (C). The numbers in the boxes of (C) and (E) are average intensities of nuclei in the yellow dashed circles.

The first enhancement step is intensity equalization. The images often had objects with high intensity such as apoptotic cells or cells during mitosis (the bright objects in Figure 2A). The intensity levels of these objects were usually 3∼5 times higher than the average intensity of the nuclei and parasites, and 10∼20 times higher than the cytoplasm signal. Furthermore, on average, the occupied area by the bright objects was at most 1% of the total image area. We used the cumulative intensity histogram of the raw images to rescale and equalize the intensity distribution. Let 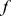 be a raw image. Based on the above observations, the algorithm calculated the intensity of higher 1% cumulative intensity level 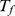 of 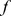 (Figure 2B), and created the intensity equalized image 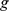 by
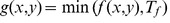
as shown in Figure 2C.

The second enhancement step is correcting the illumination bias. The images were subject to an illumination bias, which occurred during the image acquisition process (Figure 2D). The bias could be a consequence of poor physical imaging conditions such as uneven well topology, resulting in a slowly changing intensity across the image, corrupting the segmentation and parasite detection results. This artifact is well known in image processing, and we have used the illumination bias correction method previously proposed [10]:

Let 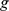 be an image, having biased illumination. Then each pixel intensity 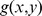 could be considered as a combination of its original intensity 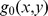 and an illumination bias artifact 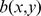, given by
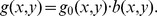



The illumination bias 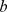 was modeled and estimated by using the Legendre polynomial. The 1-dimensional *n*-th order Legendre polynomial 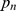 is defined by the following recurrence relation [11]:
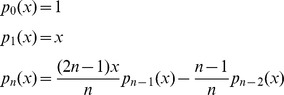
for 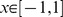. A set of 2-dimentional polynomial basis 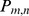 can be computed by a linear combination of 1-dimensional Legendre polynomials. Therefore, (*m*,*n*)-th order polynomial images 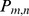 are computed given the following formula:



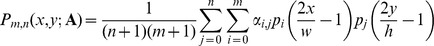
where 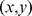 is a pixel position in 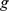, *w* and *h* are width and height of 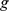 respectively, and 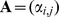 for 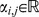 is a 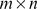 matrix.

The evaluation of 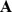 with respect to 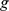 is based on a least-square minimization of the following function
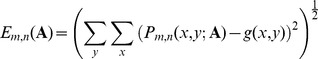
using the conjugate gradient minimization method [12]. The minimization result 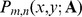 corresponds to the estimated bias 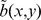 (Figure 2E), and therefore the original intensity 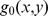 is estimated by



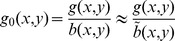
as shown in Figure 2F.

### Nucleus Region Segmentation Process

**Figure 3:**
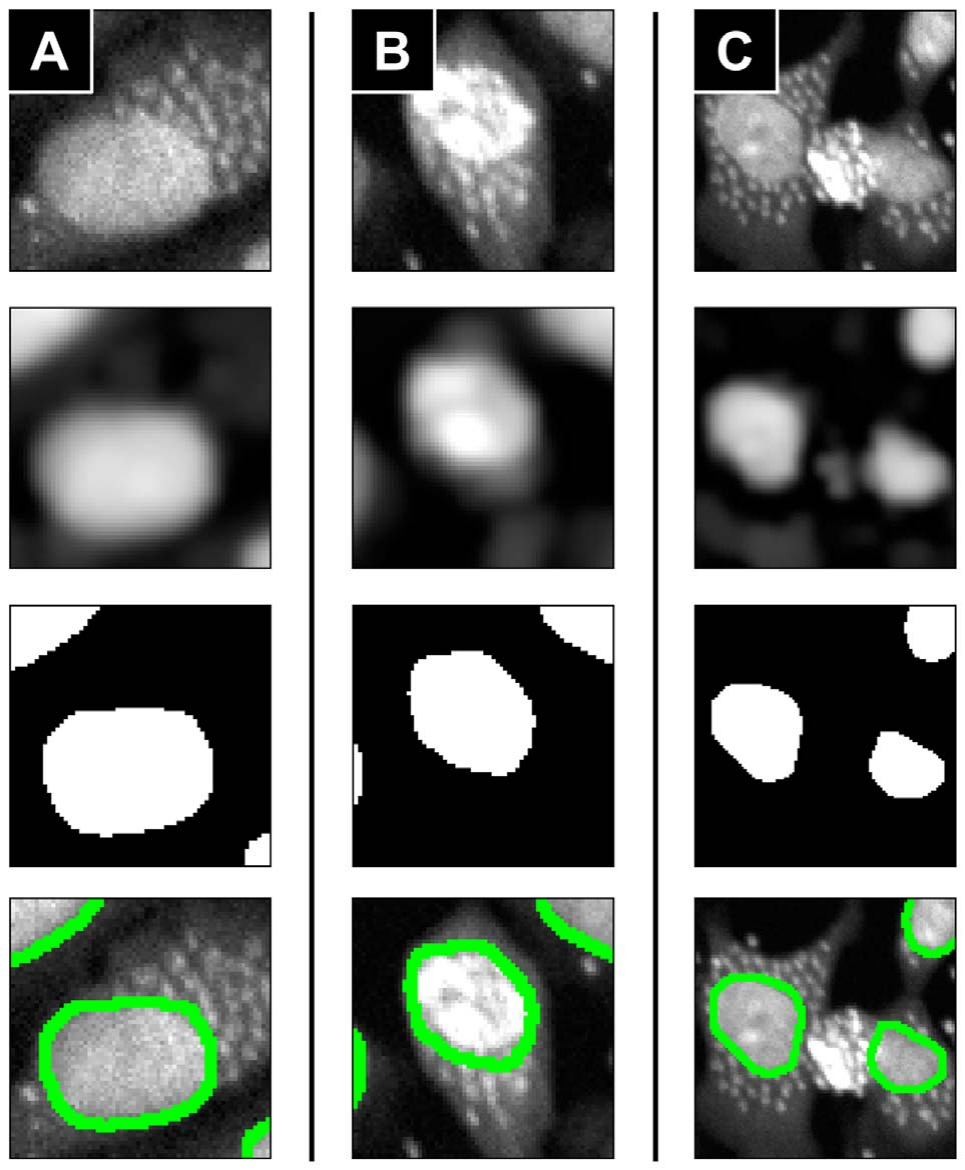
Nuclei region segmentation results for examples of difficult cases. (**A**) Parasites are too close to a nucleus. (**B**) Nucleus intensity is too high. (**C**) Parasites intensities are too high. (**Second row**) Parasite-removal images by the proposed method. (**Third row**) Nuclei masks by Otsu's thresholding method applied to the second row images. (**Fourth row**) Boundaries of segmented nuclei regions (green contours) overlapped to the original images.

The nucleus region detection was an important task because these regions were used as input in the subsequent steps of the algorithm: the individual nuclei identification, cytoplasm segmentation and parasite detection processes. In previous research, thresholding and size-based filtering, median filtering, and top-hat filtering methods have been presented to segment nuclei regions [6], [7]. However, those approaches were not suitable for our *T. cruzi* images for the following reasons:

1. Nuclei and parasites had similar intensity. Thus it was difficult to accurately segment nuclei regions by morphological filtering used in [6], [7] when parasites were closely located to nuclei (Figure 3A). The parasites may not be separated from nuclei boundaries.
2. Spatial density of parasites was higher than the images used in [6], [7], and their appearances were more blurred (Figure 3A), therefore the intensities of the regions between parasites were higher than normal cytoplasm intensity. Thus parasites may be segmented incorrectly as a part of nucleus.
3. Some nuclei and parasites had very high intensities (Figure 3B and 3C). This could also cause incorrect segmentation results.

**Figure 4:**
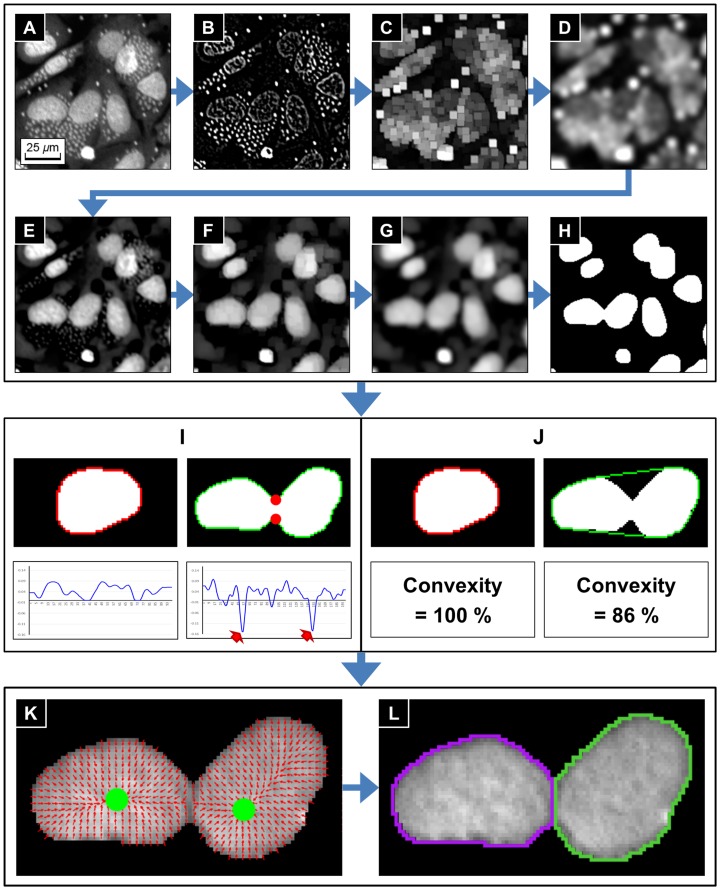
Sequential steps of individual nucleus segmentation process. (**A**) Enhanced image. (**B**) **∼** (**H**) **Nuclei region segmentation.** (**B**) Laplacian of Gaussian. (**C**) Gray-scale dilation. (**D**) Gaussian smoothing. (**E**) Subtract (D) from (A). (**F**) Gray-scale opening. (**G**) Gaussian smoothing. (**H**) Segmented nuclei regions obtained by the Otsu's thresholding method. (**I**) **∼** (**J**) **Two criteria for identifying clustered nuclei.** (**I**) Identification based on boundary curvature. (**J**) Identification based on convexity. Red arrows and dots in (I) represent local minimum curvature points and corresponding found corner points. In both (I) and (J), red contours refer isolated nucleus and green contours refer clustered nuclei. (**K**) **∼** (**L**) **Clustered nuclei splitting using GVF segmentation method.** (**K**) Gradient vector fields (red arrows) and the sink points (green dots) of clustered nuclei. (**L**) Splitting results.

In order to solve the difficulties, and to have more precisely segmented nuclei regions, we introduced a new method for nucleus region segmentation based on the discontinuity detection and morphological processing methods. The basic idea of the method was to suppress parasite signals before applying morphological operations in order to minimize the deformation of the nuclei regions. The nuclei regions were segmented by the following sequential steps:

1. Compute the Laplacian of Gaussian (LoG) of an input image (Figure 4A) in order to filter parasites signals and inner textures of nuclei (Figure 4B).
2. Apply the morphological gray-scale dilation operator to 1) in order to expand the sphere of influence of the parasite signals (Figure 4C). The structuring element is a rectangle of 7×7 pixels^2^.
3. Apply a Gaussian smoothing filter to 2) in order to have smoothed parasites sphere map (Figure 4D). The standard deviation 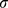 of the Gaussian smoothing kernel was chosen to be 3.
4. Subtract the parasites sphere map 3) from the original image. Then the parasites have been deformed while the nuclei keep their basic morphology (Figure 4E).
5. Apply morphological gray-scale opening operator to 4) in order to remove parasites signals completely (Figure 4F). The structuring element is a rectangle of 5×5 pixels^2^.
6. Apply Gaussian smoothing filter to 5) in order to have smooth boundaries of nuclei regions (Figure 4G). The standard deviation 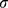 of the Gaussian smoothing is 2.
7. Separate nuclei regions by the Otsu's threshold method [13] (Figure 4H).

The size chosen for the windows and kernels in the step 2), 3), 5) and 6) were all based on observation of the average parasite size (found to be 5×5 pixels^2^). The comparison results of the previous methods and proposed method for the difficult cases are given in the result section.

### Individual Nucleus Segmentation Process

After the nuclei regions have been identified, the next step was splitting clustered nuclei into individual nuclei. This was also a crucial step because the individual nucleus was used to count total number of host cells. They were also used as the seed regions to segment the cytoplasm, and therefore have a huge impact on the final segmentation results.

We identified isolated and clustered regions, before further processing. The following two different criteria were employed to identify these clusters. The first criterion was the existence of concave corner points on the boundary. If an object was an isolated nucleus, then the object boundary should not have any concave corner points. On the other hand, if there were concave corner points on the boundary, then the object was identified as a nuclei cluster (Figure 4I). We used the osculating circle estimation method [14] to detect the concave corner points of an object boundary based on the curvature. The method can be briefly described as: For the 2-dimensional integer grid 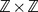, let 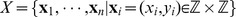 be the chain code [15] of boundary of an object, that is 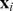 belongs to the boundary and 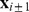 is a neighboring pixel of 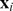. Then, the osculating circle
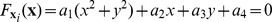
at a boundary point 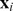 with respect to a given window size 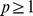 is computed by a least square minimization problem



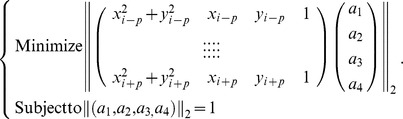



Then the center 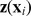 and radius 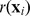 of the osculating circle at 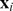 are given by
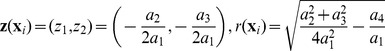
and therefore the local curvature of the boundary at 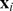 is



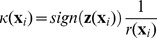
where 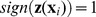 if 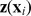 is inside of the object region, otherwise 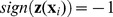. If a point on the boundary had a negative local minimum curvature (red arrows in the bottom-right plot of Figure 4I), then the point was a concave corner point (red dots in the top-right image of Figure 4I).

The second criterion used was based on the convex hull [16]. The convex hull of an object was defined as the minimal convex set of pixels containing the object. If an object was isolated nucleus, then the convex hull of the object was very close (or exactly same) to the object itself. Thus, we can identify isolated and clustered nuclei by calculating the ratio between the object area and the convex hull area, named convexity, given by
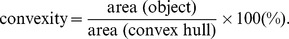



If the convexity of an object was less than a threshold, then the object was also identified as a nuclei cluster (Figure 4J). We set the threshold to 98%, which was a number obtained through various tests. If an object satisfied one of the above-mentioned criteria (concavity or convexity), then the object was identified as a cluster of nuclei.

After identification, the next step was to split the clusters into individual nuclei. For this we applied the gradient flow tracking (GFT) segmentation method [17]. The method was composed of three steps: gradient vector diffusion, gradient flow tracking, and adaptive thresholding. Let 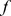 be an image and 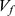 be the gradient vector field of 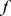 defined by
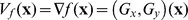
where



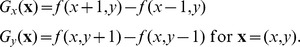



The gradients are based on first-order derivatives, hence 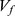 is very sensitive to noise. Therefore, 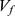 needs to be regularized before the tracking step. We used the Perona-Malik anisotropic diffusion filter [18] to diffuse 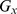 and 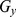 in order to suppress influence of noise and regularize gradient vectors while keeping the principle morphology of 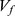. The Perona-Malik anisotropic diffusion filter is an initial value problem of the diffusion equation
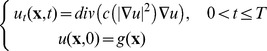
where 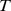 is a maximum diffusion time, and 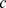 is an edge seeking function satisfies 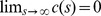 so that diffusion process stops at the location of edges. One of the most widely used edge seeking functions is



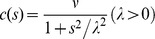
where 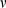 is a parameter controlling the length scale and 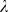 is a threshold tuning the edge seeking sensitivity. The Perona-Malik anisotropic diffusion filter is applied to 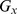 and 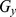 separately to diffuse 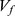. Let 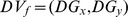 be the diffused gradient vector field. Then 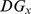 and 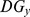 are given as the solution 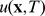 of the diffusion equation by setting the initial condition 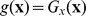 and 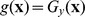 respectively.

The gradient flow tracking step is to split the individual nucleus by clustering gradient vectors. In 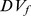 (ideally) the vectors always flow toward the sinks, which correspond to the centers of cell nuclei. To follow the gradient vectors until they stop at the sinks, the gradient flow tracking procedure is performed as following: from any starting point 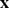, the next point 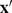 that 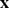 flows through in 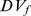 is computed as
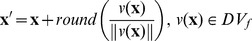
and the angle between 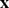 and 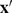 is determined as



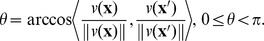



The tracking procedure is continued while 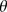 is less than 90 degrees. If 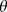 is greater than 90 degrees the tracking procedure is stopped since a sink is reached. In practice, a segmentation of the image into cell nuclei could be obtained by starting a gradient flow tracking procedure from every point in the image. The set of pixels that flow to the same sink were segmented as a nucleus. The adaptive thresholding step was to remove background regions from the segmented nuclei regions. The Otsu's thresholding method [13] was applied for the purpose.

The gradient vector field (the set of red arrows in Figure 4K) was calculated from each nuclei cluster and the gradient vectors which converged to a same sink (green dots in Figure 4K) are clustered as an individual nucleus (Figure 4L). Examples of the individual nucleus segmentation process applied to a full size images are given in Figure S4A and S4B. Most of the clustered nuclei were well separated, but the process gave wrong segmentation results when touching boundaries of nuclei were ambiguous or one of the nuclei in a cluster has strong intensity.

### Cytoplasm Segmentation Process

**Figure 5:**
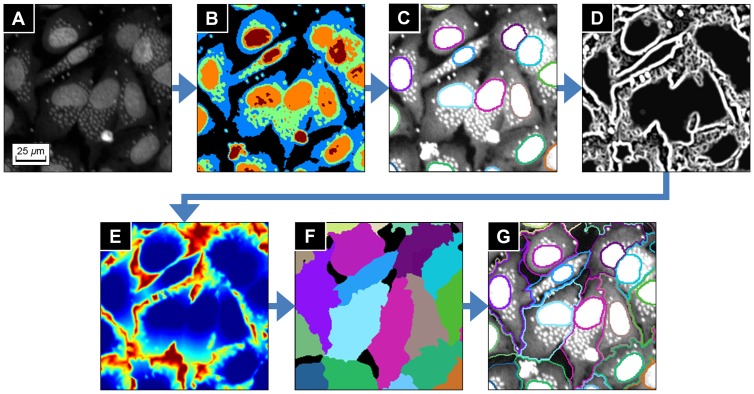
Cytoplasm segmentation using seeded cell segmentation method. (**A**) Original image. (**B**) Segmented layers by the k-means clustering segmentation. (**C**) Intensity rescaled image and previously obtained individual nuclei (seed regions for seeded cell segmentation). (**D**) Edge strength of cell regions computed by the Hessian based discontinuity detector. (**E**) Weighted distance map displayed as heat map. (**F**) Region growing result displayed with random color. (**G**) Segmentation result of the individual nuclei and cells regions.

In the images, the intensity range of the cytoplasm (300∼500) is much lower than the intensity range of nuclei and parasites (1400∼1800) as shown in Figure 5A. Therefore, it is necessary to adjust the image intensity range before segmenting cytoplasm in order to avoid the influence of the brightness difference.

To estimate the average intensity of the cytoplasm, we used the *k*-means clustering segmentation method [19]. The number of clusters *k* was chosen to be five because there are five different intensity layers: background, cytoplasm, nuclei and parasites of low intensity, middle intensity and high intensity. The average intensity 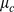 of the cytoplasm was then given by the average intensity of the second-bottom layer of the *k*-means clustering result, which is shown as blue regions in Figure 5B. In addition, the average intensity 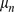 of the nuclei regions was calculated using the segmented nuclei regions produced by the nucleus region segmentation process. Then the intensity adjusted image 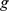 of 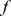 was calculated by
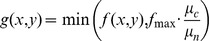
where 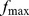 denotes the maximum intensity of 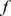 (Figure 5C).

The individual cytoplasm regions were then segmented from 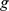 by the seeded cell segmentation method [20]. The seeded cell segmentation method was a region growing method developed for segmenting cells, and especially the irregular cytoplasm regions. The cell model used in the method assumed that the boundaries of touching cells form valley, which means that the touching boundaries are darker than inner cell regions. The region growing process started from pre-defined seed points or regions with the growing criterion given by a weighted distance, and ended when the growing regions reach to the background or other growing regions.

Let the set of seed regions 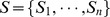 be the set of individual nuclei regions produced in the individual nucleus segmentation process (colored contours in Figure 5C). Then, let 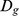 be the image domain of the image 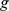, and 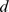 be the Euclidean distance. Then the distance from a pixel 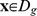 to a region 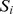 with respect to 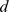 is defined by
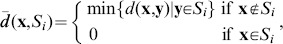
and the distance from 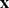 to the set of seed regions 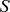 with respect to 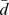 is defined by



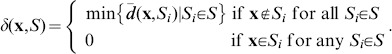



In the above 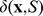 is called the Euclidean distance map of any pixel in 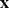 to the region 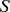.

The weighted distance used in the method was computed from the edge strength given by the Hessian matrix based discontinuity detection filter [21], [22] (Figure 5D). Let 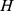 be the Hessian of the image 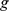, and 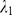, 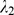 be the eigenvalues of 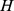 which have larger and smaller absolute values respectively. The Hessian based discontinuity detection filter 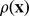 for each pixel 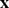 is defined by
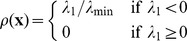
where 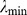 denotes the smallest eigenvalue over all pixels in the image, which in practice will always be smaller than zero. Then 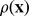 has range 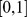, 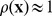 if 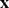 is a pixel on the discontinuity structure, and 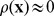 when 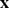 lies on a flat region. The weighted distance 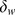 is then defined by



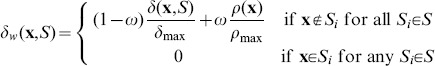
where 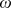 is the controlling parameter for balancing the Euclidean distance and the edge strength weight, and



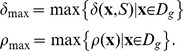



The practical implementation of the seeded cell segmentation method is as following: for the given image 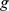 and the set of seed regions 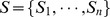,

1. Apply Gaussian smoothing filter to 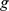 in order to reduce noise influence and regularize cell regions.
2. Segment cell regions from background by the Otsu's thresholding method [13]. Let 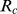 be the cell regions and 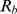 be the background regions.
3. Compute 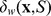 for the all pixels in 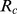 (Figure 5E).
4. Let 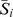 be the set of neighboring pixels of 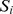 and start region growing process from each seed region 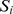.
5. For 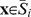, if there exists a neighborhood pixel 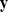 of 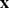 such that 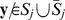 for all 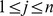, 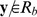 and 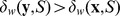, then move 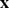 to 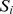, add 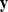 to 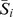, and continue the growing. If not, then move 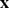 to 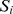 and stop growing.
6. Repeat step 5 for all 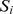 until every pixel in 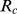 belongs to one of the seed regions (Figure 5F).

The distance map (Figure 5E) is displayed as heat map, which means the distance of a pixel is large when the color is close to red, and small when close to blue. Figure 5G shows the final cytoplasm segmentation result. Note that the parasites give no influence to the segmentation result, because the weighted distance map in the seeded cell segmentation process is based on the Hessian based discontinuity detector, and therefore spot-shape objects are suppressed when the map is generated. Examples of the cytoplasm segmentation process applied to a full size images are given in Figure S4C and S4D.

### Parasite Detection Process

**Figure 6:**
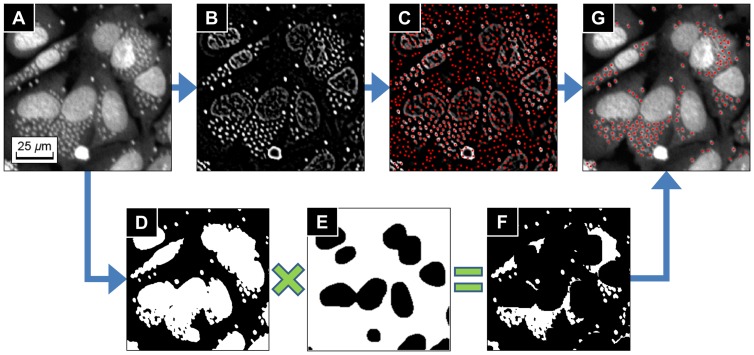
Parasite detection process. (**A**) Original image. (**B**) Laplacian of Gaussian of the original image. (**C**) Local maxima points of (B) (red dots). (**D**) Binary mask of parasites and nuclei produced from the five-class *k*-means segmentation in the cytoplasm segmentation process. (**E**) Inverse binary mask of nuclei regions from nucleus region segmentation process. (**F**) Binary mask of parasites candidates regions by multiplying (D) and (E). (**G**) Found parasites applying the binary mask (F) to the local maxima points (C).

The last part of the algorithm was parasite detection process. The parasites were detected by using the local extreme detection method [23] to the Laplacian of Gaussian of the original image, by the following sequential steps:

1. Compute Laplacian of Gaussian of an input image (Figure 6A) in order to filter parasites signals and to suppress other regional signals (Figure 6B).
2. Find local maxima points from the Laplacian of Gaussian image (red dots in Figure 6C).
3. Generate the binary mask of parasites and nuclei regions from the five-class *k*-means segmentation which is previously performed in the cytoplasm segmentation process. (Figure 6D).
4. Generate the binary mask of parasite candidate regions (Figure 6F) by multiplying 3) to the inverse binary mask of nuclei regions produced in the nucleus region segmentation process (Figure 6E).
5. Multiplying 4) to 2) to remove the local maxima points in the outside of the parasite candidate regions, and get the final parasite detection result (red dots in Figure 6G).

The *k*-means clustering segmentation used in the step 3) plays the role of sensitivity selector of the parasite detection. The parasites usually have similar intensity as the nuclei, but some of them have an intensity biased toward being darker. If we set *k* = 3 instead *k* = 2 and take the top first and second layers of the *k*-means clustering result, then the parasite candidates include those who are darker, and therefore more parasites of low intensity would be detected additionally.

### Data Analysis

From the information of segmented cytoplasm and detected parasites, the *T. cruzi* analysis algorithm labels each host cell region and assign the parasites to the host cell they belong. The algorithm outputs the following information: total number of host cells, total number of parasites, total number of infected and uninfected host cells and number of parasites for each infected host cell.

The total number of host cells was obtained by counting the individual nuclei. The total numbers of infected and uninfected host cells were obtained by counting non-empty and empty parasite labels respectively. And the number of parasites for each infected host cell was obtained by counting parasites of same label to the host cell. From those outputs, the algorithm calculated the ratio of infected host cells over the total host cells number, and average and standard deviation of the number of parasites per infected host cells which measured the anti-parasitic effects of test compounds.

## Results

The validation of the algorithm was done by 1) comparing the preciseness of our nuclei segmentation method with other methods, 2) comparing nuclei segmentation results by the algorithm with manual inspection results and 3) determining the Z' factor, infection response and EC50 from reference drug plates. The experimental assay for the algorithm validation was composed of 10 DRC plates of Benznidazole (maximum dose 400 *µ*M), 10 DRC plates of Nifurtimox (maximum dose 100 *µ*M), and 10 mock-treated plates (DMSO 1%).

### Comparison of the Nucleus Region Segmentation Methods

As previously mentioned, the precise nucleus region segmentation is an important factor in determining the reliability of the entire algorithm. We compared our nuclei region segmentation method with median filtering and top-hat filtering based methods used in previous research [6], [7]. The difficult cases images in Figure 3 were used as test images for the comparison. Those cases were 1) parasites were too close to a nucleus (3A), 2) nucleus intensity is heterogeneously high (3B), and 3) parasites intensities are abnormally high (3C). In order to ensure the fairness of the test, we used same structuring element of 7×7 pixels^2^ rectangle to those three methods. The parasites signals were removed from a test image by each method, and the nuclei regions were segmented from the images by the Otsu's thresholding method [13]. For all of the three tested images, the proposed method segmented nuclei region precisely (bottom row images in Figure 3) whereas other two methods yielded over segmentation problem (bottom row images in Figure S5).

In order to obtain a precise segmentation result, the parasite signals should be sufficiently suppressed compared to the nuclei regions. The median filtering and top-hat filtering based methods use intensity information when they performed filtering process. Thus their filtering results were strongly influenced by the nuclei or parasites of abnormally high intensity, and consequently they failed to sufficiently suppress the parasites signals as shown in Figure S5. On the other hand, our method used the Laplacian of Gaussian which is based on the neighboring intensity difference but independent to the intensity itself. Therefore, our method was less affected by the nuclei or parasites intensities, and was able to suppress the parasites signals efficiently even though parasites or nuclei had heterogeneous intensity as shown in Figure 3.

### Validation of the Nuclei Segmentation

The nuclei segmentation process was validated by comparing the algorithm segmentation results with manual inspection to the infection assay exposed to 10 different doses of reference compounds (Benznidazole and Nifurtimox). For each of the two reference compounds, four image fields were randomly selected for each dose so that in total, 80 randomly selected images were used for the validation. Table 1 shows the averaged comparison results of the Benznidazole and Nifurtimox tested assays. The entire inspection results of the 80 images are shown in Table S1 and S2. We compared each segmented nucleus by the algorithm and the manual inspection, and counted over-segmented and under-segmented nuclei. In the tables, the over-segmented is that the number of incorrectly divided nuclei by the algorithm which should not be divided, and the under-segmented is that the number of incorrectly undivided nuclei by the algorithm which should be divided. The difference is the sum of the over- and under-segmented nuclei, which implies total error of the algorithm with respect to the manual inspection results. The proportion of the difference for the algorithm compare to the manual inspection were consistently less than 5% for both the Benznidazole and the Nifurtimox images, indicating the robustness and reliability of the nuclei segmentation process.

**Table 1:** Comparison of manual and algorithm host cell nuclei detection for the test compounds.

	Benznidazole (Average±Stdev)	Nifurtimox (Average±Stdev)
Manual Count	281.23±22.50	281.65±18.44
Algorithm Count	283.55±24.75	283.35±19.38
O.Seg* (%)	6.03±2.36 (2.14±0.84%)	5.48±2.06 (1.94±0.73%)
U.Seg* (%)	3.70±2.10 (1.32±0.75%)	3.78±1.94 (1.34±0.69%)
Difference (%)	9.73±1.84 (3.46±0.66%)	9.25±2.35 (3.28±0.84%)

* O.Seg and U.Seg mean over-segmented nuclei and under-segmented nuclei respectively.

**Figure 7:**
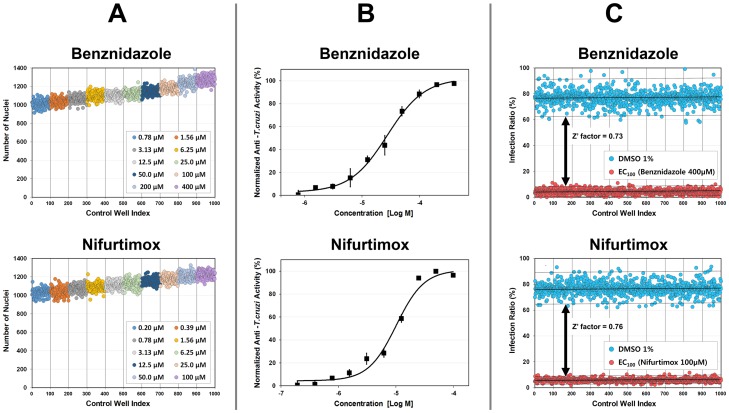
*T. cruzi* analysis algorithm validation results. Validation results for Benznidazole (top row) and Nifurtimox (bottom row) DRC plates. (**A**) Plots of the number of nuclei for 10 different concentrations. (**B**) Plots of the DRCs of normalized anti-*T.cruzi* activity. (**C**) Plots of the infection ratio of the positive control (red dots, fully uninfected) and negative control (blue dots, fully infected) wells.

Based on the stability and consistency of nuclei segmentation performance proved by the comparison with manual inspection, the nuclei segmentation performance for mass data set was validated from 1000 control wells (100 wells for each dose of 10 concentrations). Figure 7A shows the plots of the validation results and Table S3 and S4 show the average and standard deviation of the number of nuclei counted from the test images. Within a dose, the number of nuclei counted by the algorithm distributed in a narrow band (small standard deviation) around average. Across doses, the average number of nuclei forms monotonically increasing graph as dose increases. Note that the average area of cell regions became larger as the number of infected cells increased, because the cytoplasm area of infected cells became larger as parasites occupied more space in the cytoplasm. Consequently the average number of the nuclei increased as dose increased, as shown in Figure 7A, Table S3 and Table S4. Therefore the nuclei segmentation process had enough capability to provide robust and reliable data for HCS.

### EC50 and Z' factor Determination using the *T. cruzi* analysis algorithm

We determined the EC50 values from the Benznidazole and Nifurtimox DRC plates. The DRC of the drug activity against the parasites was measured by the normalized anti-*T.cruzi* activity, which was defined by the normalized ratio between number of infected host cells and number of total host cells. A four-parameter sigmoidal function
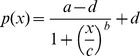
where *a* =  minimum asymptote, *b* =  slope factor, *c* =  inflection point and *d* =  maximum asymptote, and a nonlinear least square fitting method were used to calculate the DRC. The DRCs by the algorithm are shown in Figure 7B, and EC50 values obtained from the DRCs were 2.38×10^−5^ M and 8.67×10^−6^ M for Benznidazole and Nifurtimox respectively, which were within the expected ranges.

On the basis of the infection ratio from the positive controls (400 *μ*M of Benznidazole and 100 *μ*M Nifurtimox) and the negative controls (DMSO 1%), we calculated Z' factor as following equation:
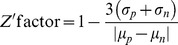
with the averages 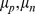 and standard deviations 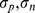 of the positive and negative controls respectively. There were clear windows between the positive and the negative controls as shown in Figure 7C, and the Z' factor were 0.73 and 0.76 for Benznidazole and Nifurtimox respectively, demonstrating the statistical confidence of the algorithm.

## Discussion

In this paper, we have presented an automated image analysis algorithm for the quantification of infection ratio and intracellular *T.cruzi* amastigote in human cell line, responded to drug activity. The most critical factors to determine image analysis is the generation of image with well distinguishing parasites and the host cell. A single DNA staining method was used for image properties to classify cells and parasites. This was achieved by the accurate segmentation and detection of both nuclei and cytoplasm of the host cells and of the parasites. The *T. cruzi* analysis algorithm first enhanced images using intensity equalization and illumination bias correction methods in order to obtain more reliable results. Nuclei regions were then segmented from background. The nucleus region segmentation process was the most important task for the entire *T. cruzi* analysis algorithm because the nuclei region mask was used for the individual nuclei identification, cytoplasm segmentation and parasite detection processes. Thresholding and size-based filtering, median filtering, and top-hat filtering methods were used in previous research [6], [7] in order to segment nuclei regions. However, those approaches were not sufficient for our analysis due to complex image conditions, such as irregular illumination and heterogeneous staining of host cells and parasites. We employed a new method for accurate nucleus region segmentation based on the discontinuity detection and morphological processing methods. As demonstrated by the comparison in the result section, the *T. cruzi* analysis algorithm showed better performance than other methods used previously [6], [7] for the difficult cases.

From the extracted nuclei regions, clustered nuclei were identified and split into individual nuclei by a gradient flow tracking segmentation. After splitting, the nuclei were counted. They were also used as the seed regions to segment cytoplasm process, and therefore had a significant influence on the final segmentation results. The individual cytoplasm regions were segmented by the seeded cell segmentation method with the individual nuclei as seed regions. Before segmenting cytoplasm, the image intensity range was adjusted in order to avoid the influence of the brightness difference of cytoplasm and nuclei regions. Since the identification and segmentation of the host cell was based simply on the DNA staining method, other types of host cells, including primary cells can be also used with great probability of success.

The parasites were detected using the local extreme detection method to the Laplacian of Gaussian of the original image, and applying the parasite region mask to the detected local maxima points. From the information of the segmented cytoplasm and detected parasites, the algorithm outputted the total number of host cells, total number of parasites, total number of infected and uninfected host cells and number of parasites for each infected host cell. The algorithm performance for HCS was validated after being compared to a manual inspection. As shown in Figure 7, the algorithm had sufficient performance to be used in HCS system for anti-trypanosomal drug discovery.

The common way to perform this type of analysis is to have cells and parasites being detected in different wavelength (image channels). We have tried this approach by using GFP-expressing parasites, however, we have observed that the infection ratio of the modified parasite was significantly decreased compared to wild type parasite, resulted in narrow windows between non-infected and infected cell. The infection ratio of wild type *T.cruzi* reaches up to average of 0.7–0.8 but for GFP-expressing parasite, the average infection ratio was only observed to be 0.3 (Figure S6). Even though using two different fluorescence fields is highly beneficial for image analysis, due to low infection ratio, GFP-expressing parasite was not applied to our image-based assay system.

Beside the application in drug discovery, this algorithm can be also used as a tool for diagnosis or any other type of study that requires detection and quantification of intracellular *T. cruzi*, saving time and increasing the precision of the process regularly performed by manual counting of the parasites on the microscope.

## Supporting Information

Figure S1 **Raw images of a negative control.** The original images of Figure 1A. A 16-bit image viewer is recommended for image reading.
(TIF)

Figure S2 **Raw images of a positive control.** The original images of Figure 1B. A 16-bit image viewer is recommended for image reading.
(TIF)

Figure S3 ***T. cruzi*** **analysis algorithm diagram.** The algorithm outputs analysis data from input images by following five sequential processes: image enhancement, nucleus region segmentation, individual nucleus segmentation, cytoplasm segmentation and parasite detection.
(TIF)

Figure S4 **Examples of individual nuclei and cytoplasm segmentation process.** Individual nuclei segmentation results of (**A**) Negative control image. (**B**) Positive control image. Cytoplasm segmentation results of (**C**) Negative control image. (**D**) Positive control image.
(TIF)

Figure S5 **Nuclei region segmentation results of the median and top-hat filtering based methods applied to the example images in** **Figure 3** **.** (**A**) Results of the median filtering based method with window size of 7×7 pixels^2^. (**B**) Results of the top-hat filtering based method with window size of 7×7 pixels^2^. (**First row**) Parasite-removal images by the methods. (**Second row**) Nuclei masks by Otsu's thresholding method applied to the top row images. (**Third row**) Boundaries of segmented nuclei regions (green contours) overlapped to the original images.
(TIF)

Figure S6 **Infection ratio of GFP tagged T. cruzi in U2OS cell line.** (**A**) Images for GFP-expressing *T. cruzi* in host cell. Red signal is DNA staining and Green signal is GFP-expressing *T. cruzi*. (B) Plots of the infection ratio of 256 control wells (fully infected). The average infection ratio was 30.66±5.93%.
(TIF)

Table S1 **Comparison of manual and algorithm host cell nuclei detection for Benznidazole DRC plates.**
(PDF)

Table S2 **Comparison of manual and algorithm host cell nuclei detection for Nifurtimox DRC plates.**
(PDF)

Table S3 **Number of host cells counted from Benznidazole DRC plates.**
(PDF)

Table S4 **Number of host cells counted from Nifurtimox DRC plates.**
(PDF)
