# Supplementary figures and images for: An Image-Based Algorithm for Precise and Accurate High Throughput Assessment of Drug Activity against the Human Parasite Trypanosoma cruzi
Source: PLoS One. 2014 Feb 4;9(2):e87188. doi: 10.1371/journal.pone.0087188 (PMC3913590; doi:10.1371/journal.pone.0087188)

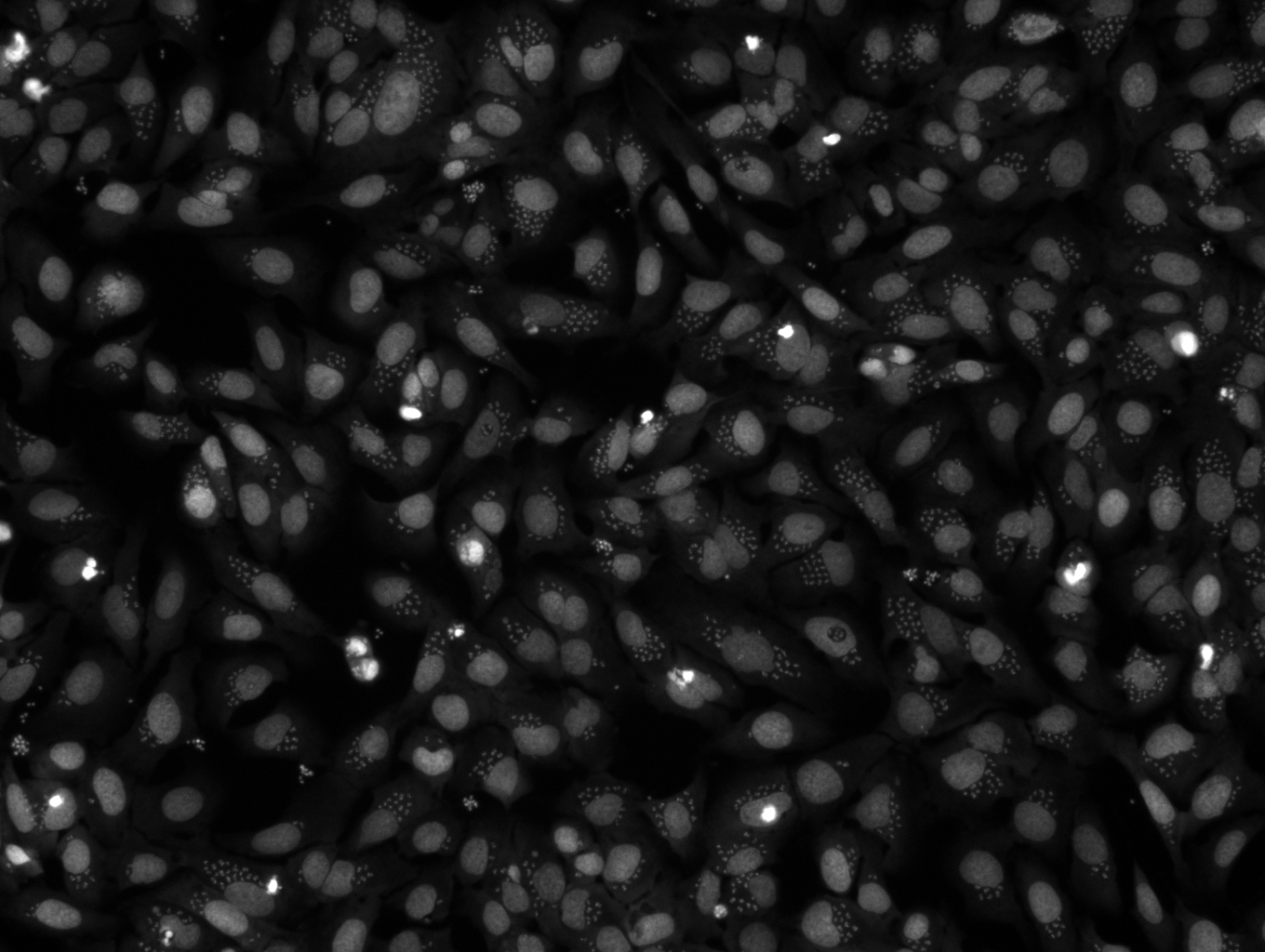

Supplement: Figure S1 — Raw images of a negative control. The original images of Figure 1A. A 16-bit image viewer is recommended for image reading. (TIF) [file pone.0087188.s001.tif]

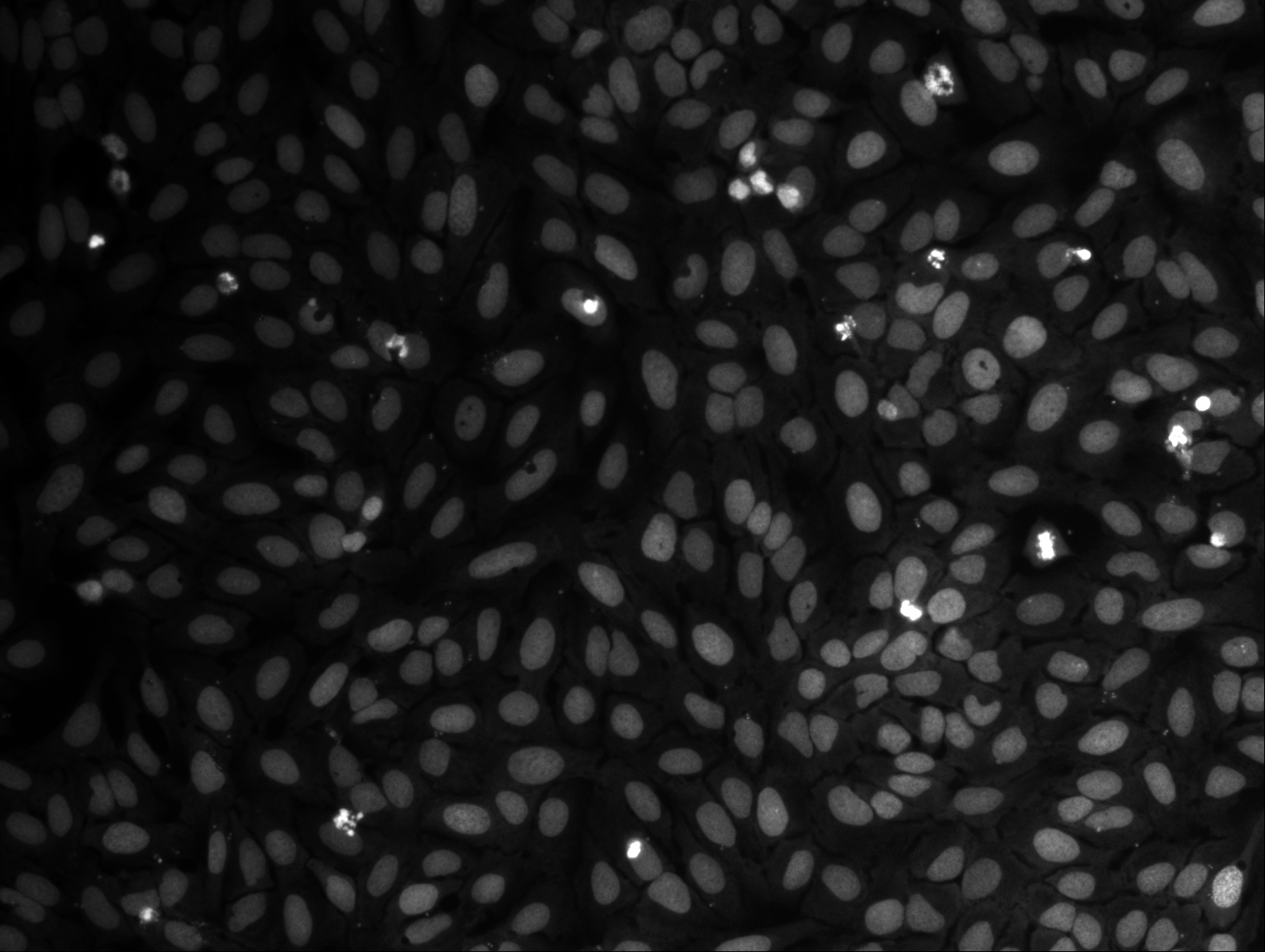

Supplement: Figure S2 — Raw images of a positive control. The original images of Figure 1B. A 16-bit image viewer is recommended for image reading. (TIF) [file pone.0087188.s002.tif]

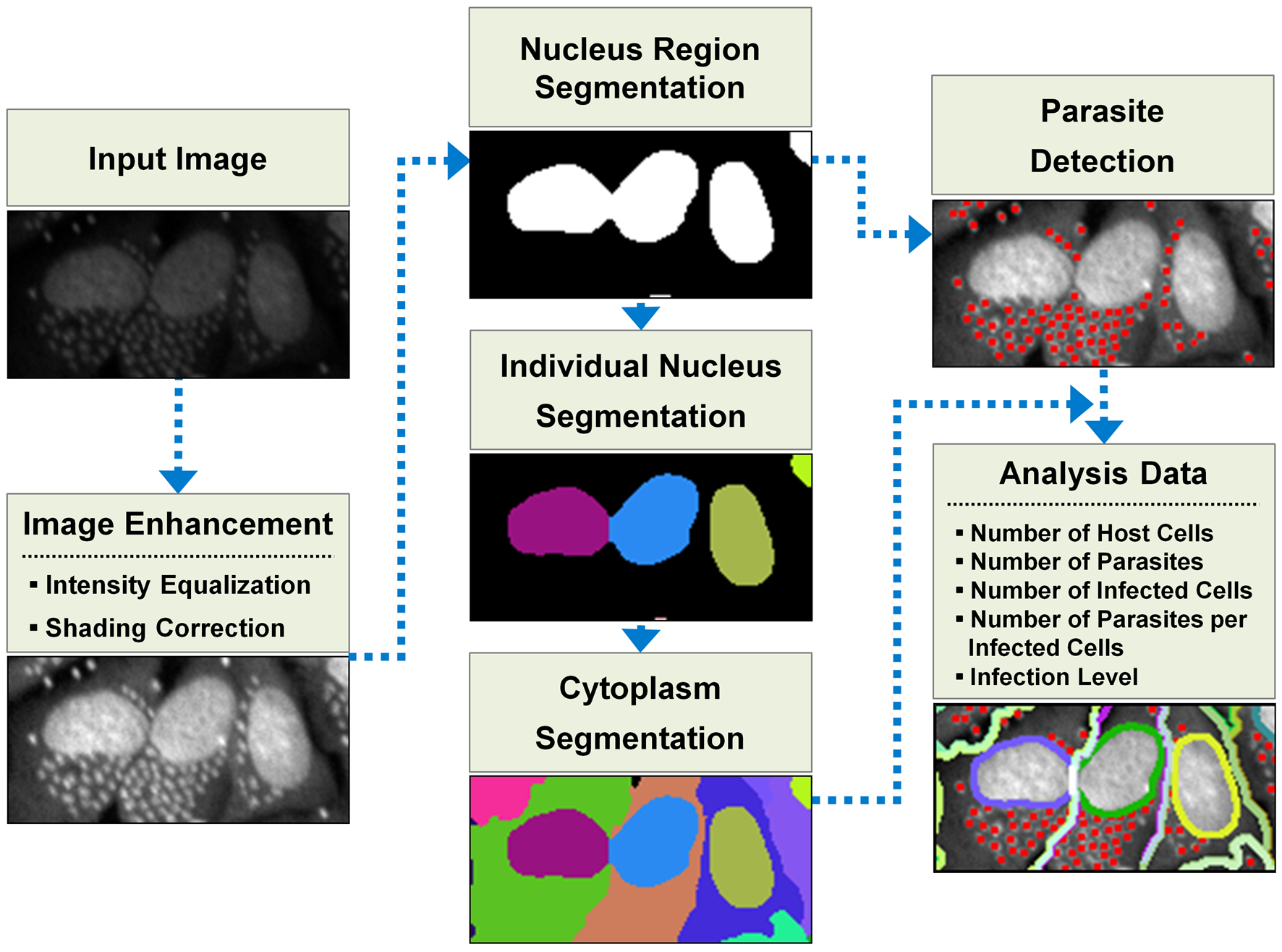

Supplement: Figure S3 — T. cruzi analysis algorithm diagram. The algorithm outputs analysis data from input images by following five sequential processes: image enhancement, nucleus region segmentation, individual nucleus segmentation, cytoplasm segmentation and parasite detection. (TIF) [file pone.0087188.s003.tif]

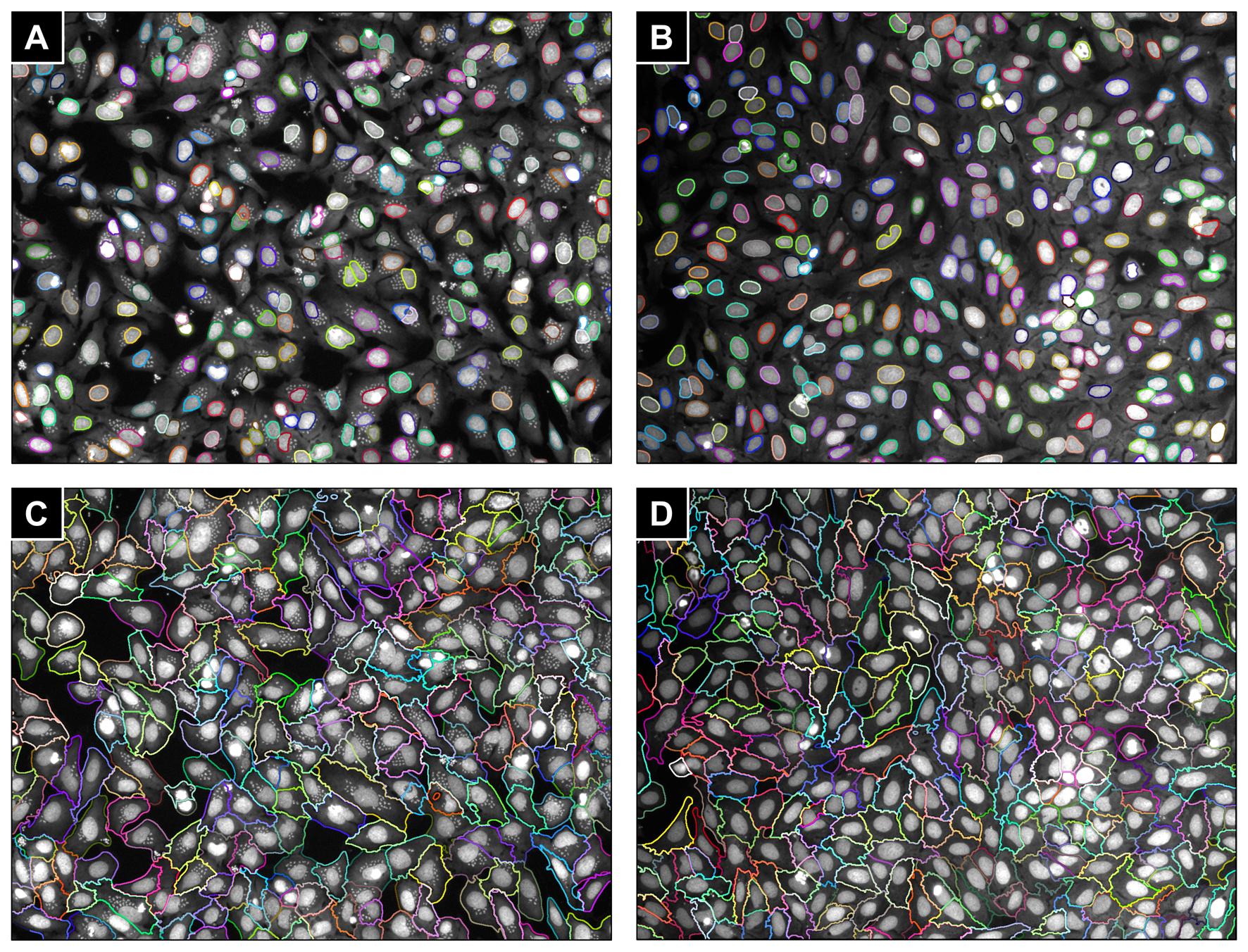

Supplement: Figure S4 — Examples of individual nuclei and cytoplasm segmentation process. Individual nuclei segmentation results of (A) Negative control image. (B) Positive control image. Cytoplasm segmentation results of (C) Negative control image. (D) Positive control image. (TIF) [file pone.0087188.s004.tif]

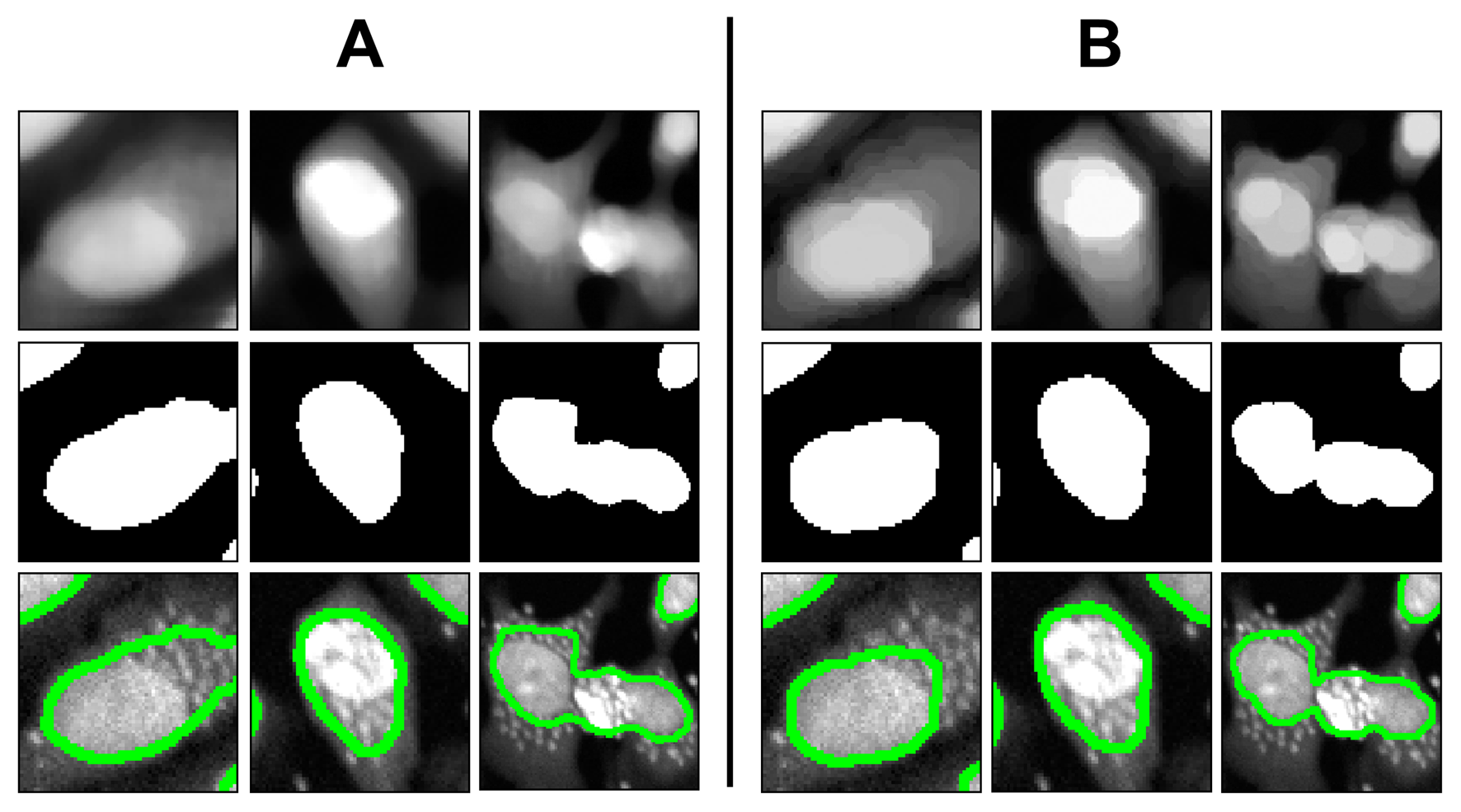

Supplement: Figure S5 — Nuclei region segmentation results of the median and top-hat filtering based methods applied to the example images in Figure 3 . (A) Results of the median filtering based method with window size of 7×7 pixels2. (B) Results of the top-hat filtering based method with window size of 7×7 pixels2. (First row) Parasite-removal images by the methods. (Second row) Nuclei masks by Otsu's thresholding method applied to the top row images. (Third row) Boundaries of segmented nuclei regions (green contours) overlapped to the original images. (TIF) [file pone.0087188.s005.tif]

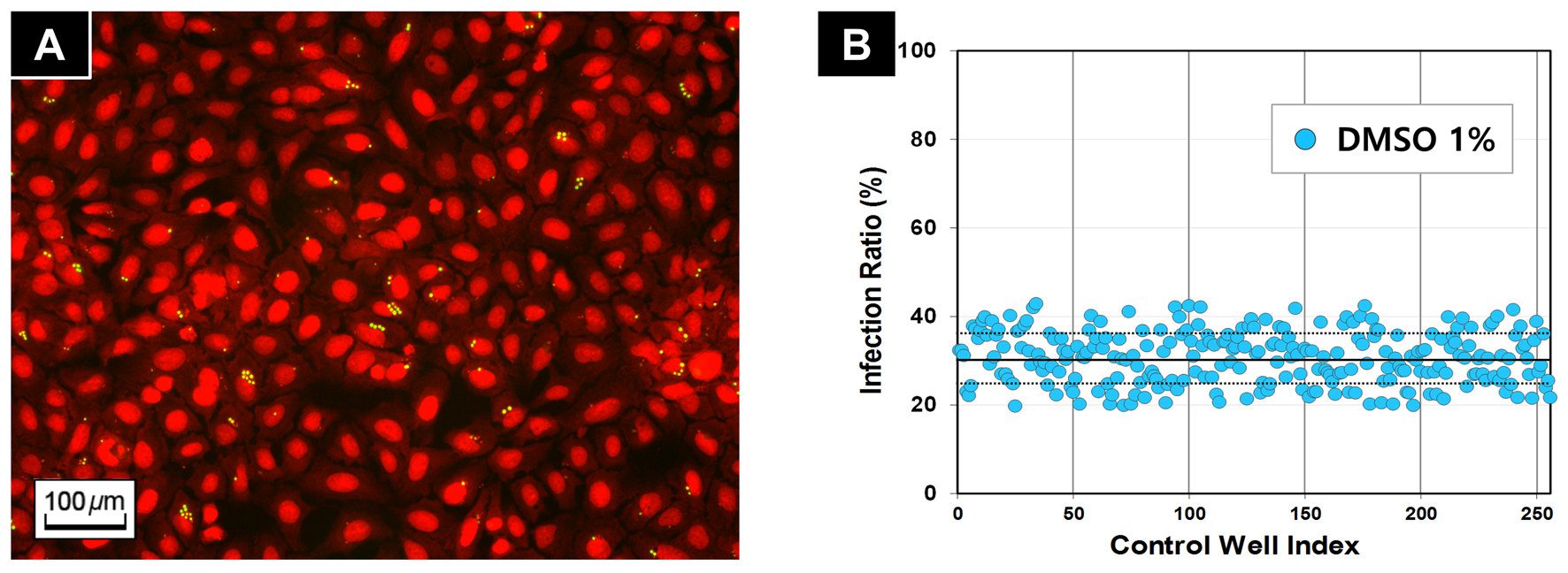

Supplement: Figure S6 — Infection ratio of GFP tagged T. cruzi in U2OS cell line. (A) Images for GFP-expressing T. cruzi in host cell. Red signal is DNA staining and Green signal is GFP-expressing T. cruzi. (B) Plots of the infection ratio of 256 control wells (fully infected). The average infection ratio was 30.66±5.93%. (TIF) [file pone.0087188.s006.tif]
