# Supplementary material for: An Image-Based Algorithm for Precise and Accurate High Throughput Assessment of Drug Activity against the Human Parasite Trypanosoma cruzi
Source: PLoS One. 2014 Feb 4;9(2):e87188. doi: 10.1371/journal.pone.0087188 (PMC3913590; doi:10.1371/journal.pone.0087188)
Supplement: Table S1 — Comparison of manual and algorithm host cell nuclei detection for Benznidazole DRC plates. (PDF) [file pone.0087188.s007.pdf]

Table S1. Comparison of manual and algorithm host cell nuclei detection for Benznidazole DRC plates.

		Manual	Algorithm	O.Seg* (%)	U.Seg* (%)	Difference (%)
0.78 µM	Image 1	243	245	5 (2.06 %)	3 (1.23 %)	8 (3.29 %)
	Image 2	262	265	7 (2.67 %)	4 (1.53 %)	11 (4.20 %)
	Image 3	251	249	4 (1.59 %)	6 (2.39 %)	10 (3.98 %)
	Image 4	235	238	6 (2.55 %)	3 (1.28 %)	9 (3.83 %)
1.56 µM	Image 1	258	252	1 (0.39 %)	7 (2.71 %)	8 (3.10 %)
	Image 2	255	250	2 (0.78 %)	7 (2.75 %)	9 (3.53 %)
	Image 3	263	261	5 (1.90 %)	7 (2.66 %)	12 (4.56 %)
	Image 4	279	286	8 (2.87 %)	1 (0.36 %)	9 (3.23 %)
3.13 µM	Image 1	280	281	7 (2.50 %)	6 (2.14 %)	13 (4.64 %)
	Image 2	260	266	9 (3.46 %)	3 (1.15 %)	12 (4.62 %)
	Image 3	252	251	2 (0.79 %)	3 (1.19 %)	5 (1.98 %)
	Image 4	273	270	3 (1.10 %)	6 (2.20 %)	9 (3.30 %)
6.25 µM	Image 1	272	275	6 (2.21 %)	3 (1.10 %)	9 (3.31 %)
	Image 2	289	288	5 (1.73 %)	6 (2.08 %)	11 (3.81 %)
	Image 3	263	262	4 (1.52 %)	5 (1.90 %)	9 (3.42 %)
	Image 4	274	273	3 (1.09 %)	4 (1.46 %)	7 (2.55 %)
12.5 µM	Image 1	281	286	9 (3.20 %)	4 (1.42 %)	13 (4.63 %)
	Image 2	278	279	4 (1.44 %)	3 (1.08 %)	7 (2.52 %)
	Image 3	281	280	4 (1.42 %)	5 (1.78 %)	9 (3.20 %)
	Image 4	259	255	3 (1.16 %)	7 (2.70 %)	10 (3.86 %)
25.0 µM	Image 1	281	277	4 (1.42 %)	8 (2.85 %)	12 (4.27 %)
	Image 2	275	271	3 (1.09 %)	7 (2.55 %)	10 (3.64 %)
	Image 3	264	269	7 (2.65 %)	2 (0.76 %)	9 (3.41 %)
	Image 4	296	301	8 (2.70 %)	3 (1.01 %)	11 (3.72 %)
50.0 µM	Image 1	289	295	8 (2.77 %)	2 (0.69 %)	10 (3.46 %)
	Image 2	277	281	7 (2.53 %)	3 (1.08 %)	10 (3.61 %)
	Image 3	274	279	7 (2.55 %)	2 (0.73 %)	9 (3.28 %)
	Image 4	301	304	7 (2.33 %)	4 (1.33 %)	11 (3.65 %)
100 µM	Image 1	285	292	8 (2.81 %)	1 (0.35 %)	9 (3.16 %)
	Image 2	298	302	7 (2.35 %)	3 (1.01 %)	10 (3.36 %)
	Image 3	287	294	9 (3.14 %)	2 (0.70 %)	11 (3.83 %)
	Image 4	314	322	9 (2.87 %)	1 (0.32 %)	10 (3.18 %)
200 µM	Image 1	300	308	10 (3.33 %)	2 (0.67 %)	12 (4.00 %)
	Image 2	302	305	6 (1.99 %)	3 (0.99 %)	9 (2.98 %)
	Image 3	326	332	7 (2.15 %)	1 (0.31 %)	8 (2.45 %)
	Image 4	304	311	8 (2.63 %)	1 (0.33 %)	9 (2.96 %)
400 µM	Image 1	306	316	10 (3.27 %)	0 (0.00 %)	10 (3.27 %)
	Image 2	335	339	9 (2.69 %)	5 (1.49 %)	14 (4.18 %)
	Image 3	310	314	5 (1.61 %)	1 (0.32 %)	6 (1.94 %)
	Image 4	317	318	5 (1.58 %)	4 (1.26 %)	9 (2.84 %)
Average±Stdev		281.23±22.50	283.55±24.75	6.03±2.36	3.70±2.10	9.73±1.84
		-	-	(2.14±0.84 %)	(1.32±0.75 %)	(3.46±0.66 %)

* O.Seg and U.Seg mean over-segmented nuclei and under-segmented nuclei respectively.
