# Supplementary material for: An Image-Based Algorithm for Precise and Accurate High Throughput Assessment of Drug Activity against the Human Parasite Trypanosoma cruzi
Source: PLoS One. 2014 Feb 4;9(2):e87188. doi: 10.1371/journal.pone.0087188 (PMC3913590; doi:10.1371/journal.pone.0087188)
Supplement: Table S2 — Comparison of manual and algorithm host cell nuclei detection for Nifurtimox DRC plates. (PDF) [file pone.0087188.s008.pdf]

Table S2. Comparison of manual and algorithm host cell nuclei detection for Nifurtimox DRC plates.

		Manual	Algorithm	O.Seg* (%)	U.Seg* (%)	Difference (%)
0.20 µM	Image 1	253	251	4 (1.58 %)	6 (2.37 %)	10 (3.95 %)
	Image 2	264	264	4 (1.52 %)	4 (1.52 %)	8 (3.03 %)
	Image 3	247	246	4 (1.62 %)	5 (2.02 %)	9 (3.64 %)
	Image 4	262	264	7 (2.67 %)	5 (1.91 %)	12 (4.58 %)
0.39 µM	Image 1	257	254	4 (1.56 %)	7 (2.72 %)	11 (4.28 %)
	Image 2	259	261	5 (1.93 %)	3 (1.16 %)	8 (3.09 %)
	Image 3	278	275	2 (0.72 %)	5 (1.80 %)	7 (2.52 %)
	Image 4	266	269	6 (2.26 %)	3 (1.13 %)	9 (3.38 %)
0.78 µM	Image 1	268	269	4 (1.49 %)	3 (1.12 %)	7 (2.61 %)
	Image 2	280	281	6 (2.14 %)	5 (1.79 %)	11 (3.93 %)
	Image 3	256	259	4 (1.56 %)	1 (0.39 %)	5 (1.95 %)
	Image 4	271	274	5 (1.85 %)	2 (0.74 %)	7 (2.58 %)
1.56 µM	Image 1	265	263	3 (1.13 %)	5 (1.89 %)	8 (3.02 %)
	Image 2	262	263	3 (1.15 %)	2 (0.76 %)	5 (1.90 %)
	Image 3	288	290	7 (2.43 %)	5 (1.74 %)	12 (4.17 %)
	Image 4	281	280	5 (1.78 %)	6 (2.14 %)	11 (3.91 %)
3.13 µM	Image 1	263	268	6 (2.28 %)	1 (0.38 %)	7 (2.66 %)
	Image 2	290	294	7 (2.41 %)	3 (1.03 %)	10 (3.45 %)
	Image 3	271	267	4 (1.48 %)	8 (2.95 %)	12 (4.43 %)
	Image 4	282	286	9 (3.19 %)	5 (1.77 %)	14 (4.96 %)
6.25 µM	Image 1	271	277	8 (2.95 %)	2 (0.74 %)	10 (3.69 %)
	Image 2	292	294	5 (1.71 %)	3 (1.03 %)	8 (2.74 %)
	Image 3	265	266	6 (2.26 %)	5 (1.88 %)	11 (4.15 %)
	Image 4	286	289	6 (2.10 %)	3 (1.05 %)	9 (3.15 %)
12.5 µM	Image 1	293	298	8 (2.73 %)	3 (1.02 %)	11 (3.75 %)
	Image 2	275	280	7 (2.55 %)	2 (0.73 %)	9 (3.27 %)
	Image 3	304	309	7 (2.30 %)	2 (0.66 %)	9 (2.96 %)
	Image 4	286	283	5 (1.75 %)	8 (2.80 %)	13 (4.55 %)
25.0 µM	Image 1	300	304	7 (2.33 %)	3 (1.00 %)	10 (3.33 %)
	Image 2	295	304	9 (3.05 %)	0 (0.00 %)	9 (3.05 %)
	Image 3	306	305	4 (1.31 %)	5 (1.63 %)	9 (2.94 %)
	Image 4	284	284	5 (1.76 %)	5 (1.76 %)	10 (3.52 %)
50.0 µM	Image 1	298	298	4 (1.34 %)	4 (1.34 %)	8 (2.68 %)
	Image 2	314	310	1 (0.32 %)	5 (1.59 %)	6 (1.91 %)
	Image 3	299	305	8 (2.68 %)	2 (0.67 %)	10 (3.34 %)
	Image 4	303	305	3 (0.99 %)	1 (0.33 %)	4 (1.32 %)
100 µM	Image 1	301	302	3 (1.00 %)	2 (0.66 %)	5 (1.66 %)
	Image 2	317	315	5 (1.58 %)	7 (2.21 %)	12 (3.79 %)
	Image 3	309	317	10 (3.24 %)	2 (0.65 %)	12 (3.88 %)
	Image 4	305	311	9 (2.95 %)	3 (0.98 %)	12 (3.93 %)
Average±Stdev		281.65±18.44	283.35±19.38	5.48±2.06	3.78±1.94	9.25±2.35
		-	-	(1.94±0.73 %)	(1.34±0.69 %)	(3.28±0.84 %)

* O.Seg and U.Seg mean over-segmented nuclei and under-segmented nuclei respectively.
