# Supplementary material for: An Image-Based Algorithm for Precise and Accurate High Throughput Assessment of Drug Activity against the Human Parasite Trypanosoma cruzi
Source: PLoS One. 2014 Feb 4;9(2):e87188. doi: 10.1371/journal.pone.0087188 (PMC3913590; doi:10.1371/journal.pone.0087188)
Supplement: Table S3 — Number of host cells counted from Benznidazole DRC plates. (PDF) [file pone.0087188.s009.pdf]

Table S3. Number of host cells counted from Benznidazole DRC plates.

	Number of host cells	
Dose	Average	Stdev
0.78 μM	1012.35	39.72
1.56 μM	1035.59	35.27
3.13 μM	1061.03	36.66
6.25 μM	1095.08	40.95
12.5 μM	1102.52	34.38
25.0 μM	1122.57	37.55
50.0 μM	1144.02	36.10
100 μM	1181.30	35.24
200 μM	1233.54	45.60
400 μM	1268.36	42.49
