# Supplementary material for: An Image-Based Algorithm for Precise and Accurate High Throughput Assessment of Drug Activity against the Human Parasite Trypanosoma cruzi
Source: PLoS One. 2014 Feb 4;9(2):e87188. doi: 10.1371/journal.pone.0087188 (PMC3913590; doi:10.1371/journal.pone.0087188)
Supplement: Table S4 — Number of host cells counted from Nifurtimox DRC plates. (PDF) [file pone.0087188.s010.pdf]

Table S4. Number of host cells counted from Nifurtimox DRC plates.

	Number of host cells	
Dose	Average	Stdev
0.20 μM	1027.82	40.24
0.39 μM	1035.70	48.91
0.78 μM	1071.71	39.00
1.56 μM	1089.08	40.14
3.13 μM	1106.22	36.73
6.25 μM	1122.66	46.10
12.5 μM	1154.59	39.18
25.0 μM	1177.26	36.84
50.0 μM	1207.21	40.60
100 μM	1222.19	38.62
